# Neuropsychiatric Systemic Lupus Erythematosus: A Case Series

**DOI:** 10.7759/cureus.99634

**Published:** 2025-12-19

**Authors:** Manuel A Garcia-Smester, Asha Patnaik

**Affiliations:** 1 Rheumatology, Maimonides Medical Center, Brooklyn, USA; 2 Rheumatology, Stony Brook University, Stony Brook, USA

**Keywords:** neuropsychiatric lupus, neuropsychiatric sle, neuropsychiatric systemic lupus erythematosus (npsle), pathogenesis, systemic lupus erythematosus

## Abstract

Neuropsychiatric systemic lupus erythematosus (NPSLE) is a rare and poorly understood manifestation of systemic lupus erythematosus, encompassing a wide range of neurological and psychiatric symptoms. In order to highlight the diagnostic complexity and therapeutic challenges of NPSLE, this report presents the management of three distinct presentations. We retrospectively reviewed cases of three female patients with NPSLE admitted to a tertiary care hospital. Clinical presentation, diagnostic workup, treatment strategies, and outcomes were analyzed. Each case demonstrated unique NPSLE manifestations: acute confusional state in the setting of sepsis, cerebral vasculitis with retinal involvement, and catatonia. Treatments included high-dose corticosteroids, cyclophosphamide, and mycophenolate mofetil, with varying degrees of clinical improvement. NPSLE remains a diagnostic challenge due to its heterogeneity and lack of definitive biomarkers. Early recognition and individualized immunosuppressive treatment are essential for improved outcomes.

## Introduction

Neuropsychiatric systemic lupus erythematosus (NPSLE) is a rare and poorly understood manifestation of systemic lupus erythematosus (SLE), an autoimmune disorder that can affect multiple major organ systems. The nervous system, while rare, can be also affected. Manifestations of SLE in the nervous system can be diverse. The American College of Rheumatology (ACR) recognizes 19 distinct NPSLE syndromes, which may affect either the central or peripheral nervous system [1]. These symptoms can range from subtle presentations, such as headache, cognitive dysfunction, mood disturbances, or anxiety, to more severe conditions, such as myelopathy, autonomic neuropathy, demyelinating syndromes, and cerebrovascular disease [2]. Treatment varies depending on the specific manifestation and severity. Diagnosing NPSLE remains challenging due to coexisting comorbidities, and laboratory findings can often be inconclusive. Hence, early recognition through thorough history-taking is critical [3]. In this case series, we present three distinct presentations of NPSLE and their respective treatment approaches, in hopes of guiding future management for clinicians.

## Case presentation

Case 1

**Figure 1:**
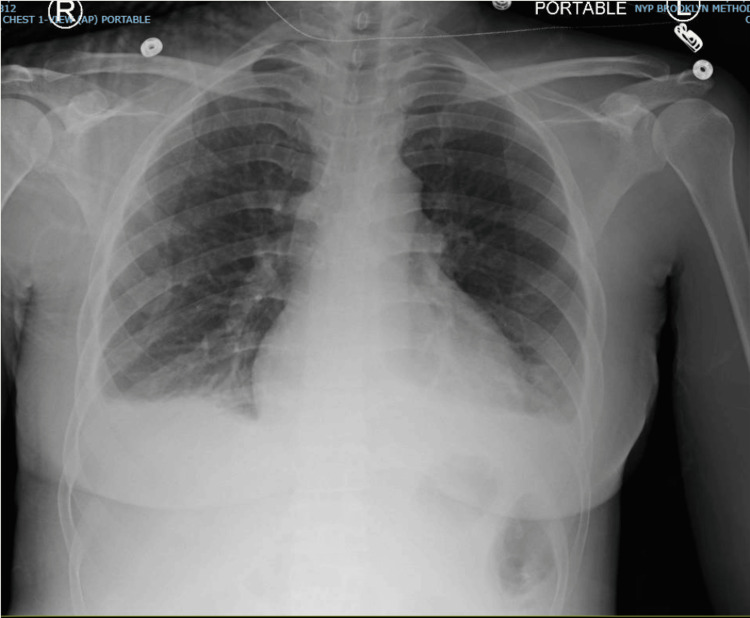
Chest X-ray of case 1 showing right lower lobe consolidation

A 36-year-old African American female patient with a known history of SLE (not following with a rheumatologist and off medication for years) presented with a several-day history of fever, malaise, cough, arthralgia, and myalgia. She was febrile and tachycardic and had leukocytosis (white blood cell [WBC] count 21 K/UL). Chest X-ray revealed right lower lobe consolidation, consistent with sepsis secondary to focal pneumonia (Figure 1). She was started on antibiotics, and her condition initially improved.

However, on hospital day 3, she developed acute confusion, hallucinations (describing creatures crawling up the walls), and worsening mental status, eventually requiring intubation. Neurology and Infectious Disease were consulted. CT of the head was unremarkable, MRI of the brain was unremarkable, EEG was negative, and antibiotics were adjusted for possible medication-induced encephalopathy. Though she was successfully extubated, her mental status remained impaired despite radiographic resolution of pneumonia.

Rheumatology was consulted due to her SLE history. Labs, as seen in Table 1, revealed mildly elevated dsDNA, positive anti-Smith antibodies, positive RNP antibodies, and hypocomplementemia. CSF analysis showed elevated protein, increased oligoclonal bands, and elevated IgG synthesis rate; cultures were negative, and further CSF studies ruled out any infectious source, with full viral CSF panel including Venereal Disease Research Lab test (VDRL), varicella zoster virus, herpes simplex virus, Ebstein-Barr Virus, cytomegalovirus, rapid plasmin reagin, and JC virus all negative. This raised suspicion for lupus psychosis induced by NPSLE, and she was started on pulse-dose steroids. After minimal improvement by day 3, cyclophosphamide was initiated per the NIH protocol, which consists of intravenous cyclophosphamide (0.5-1 gm/m^2^, adjusted to WBC nadir, given monthly for six months [4]. Following this and a five-day course of pulse-dose steroids, her mental status significantly improved. She completed six months of cyclophosphamide with a prolonged steroid taper, started hydroxychloroquine, and has remained clinically stable off steroids since.

**Table 1:** Antibody profile of case 1 ANA, anti-nuclear antibody; dsDNA, double-stranded DNA; SSA, Sjögren's syndrome-related antibody A; SSB, Sjögren's-syndrome-related antigen B antibody; Sm, Smith; RNP, ribonucleoprotein

Parameter	Patient Value	Reference Range
ANA	Positive	-
dsDNA	7 AU	<5 AU
Anti-SSA	<1.0 AU	<1 AU
Anti-SSB	<1.0 AU	<1 AU
Anti-Sm	>8.0 AU	<1 AU
Anti- RNP	>8.0 AU	<1 AU
C3	55 mg/dL	<84 AU
C4	14 mg/dL	<15 AU

Case 2

A 32-year-old African American female patient with no prior medical history presented with worsening abdominal pain, nausea, and vomiting for one week. She had previously visited an outside hospital for similar complaints and was told she had abdominal lymphadenopathy. She also reported a chronic malar rash for over two years and progressive vision loss over the prior two weeks.

**Figure 2:**
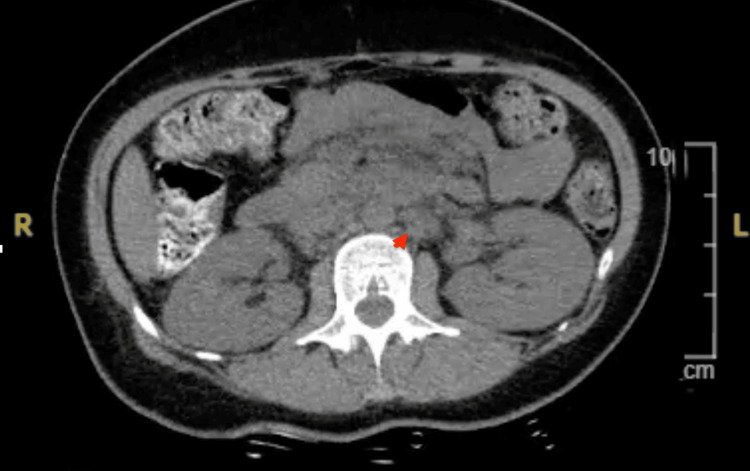
CT of the abdomen and pelvis with significant retroperitoneal lymphadenopathy in case 2

**Figure 3:**
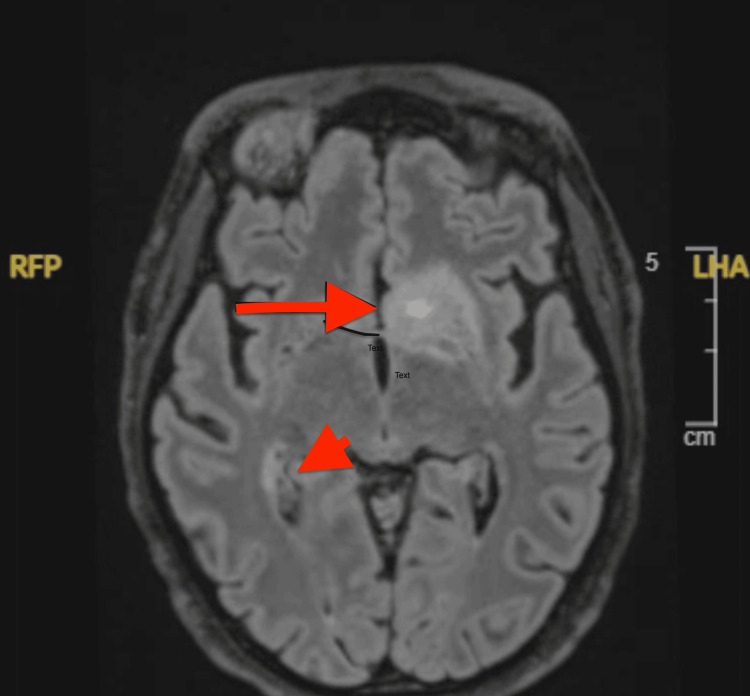
MRI of the brain showing infarction in case 2

Examination revealed hyperpigmented, scaly plaques on the cheeks and nose sparing the nasolabial folds, along with periorbital edema and reduced vision. Imaging showed extensive mesenteric and retroperitoneal lymphadenopathy (Figure 2). Labs showed pancytopenia and positive ANA, anti-Smith, anti-RNP, and anti-SS-A antibodies (Table 2). Ophthalmology noted retinal vasculitis, and MRI of the brain revealed multiple infarcts (Figure 3). CSF showed elevated protein and IgG synthesis rate. Infectious workup was negative, and MRA showed no vascular occlusion.

**Table 2:** Antibody panel profile of case 2 ANA, anti-nuclear antibody; dsDNA, double-stranded DNA; SSA, Sjögren's syndrome-related antibody A; SSB, Sjögren's-syndrome-related antigen B antibody; Sm, Smith; RNP, ribonucleoprotein

Parameter	Patient Value	Reference Range
ANA	Positive	-
dsDNA	19 AU	<5 AU
Anti-SSA	5.7 AU	<1 AU
Anti-SSB	<1.0 AU	<1 AU
Anti-Sm	>8.0 AU	<1 AU
Anti- RNP	>8.0 AU	<1 AU
C3	31 mg/dL	>84 AU
C4	8 mg/dL	>15 AU

Lupus-induced cerebral vasculitis was suspected. The patient was treated with pulse-dose steroids and cyclophosphamide per NIH protocol [1], with good clinical response. She is currently in remission and maintained on azathioprine after completing cyclophosphamide therapy and a prolonged steroid taper.

Case 3

A 38-year-old Bengali female patient with a history of pituitary adenoma, leiomyoma (status post-partial hysterectomy), and pelvic inflammatory disease presented with a two-day history of hallucinations. She had recently established rheumatologic care for a positive ANA, facial rash, and photosensitivity. Further evaluation revealed positive anti-Smith and anti-RNP antibodies, with low complement (Table 3).

**Table 3:** Antibody panel profile of case 3 ANA, anti-nuclear antibody; dsDNA, double-stranded DNA; SSA, Sjögren's syndrome-related antibody A; SSB, Sjögren's-syndrome-related antigen B antibody; Sm, Smith; RNP, ribonucleoprotein

Parameter	Patient Value	Reference Range
ANA	Positive	-
dsDNA	<5 AU	<5 AU
Anti-SSA	<1.0 AU	<1 AU
Anti-SSB	<1.0 AU	<1 AU
Anti-Sm	2.3 AU	<1 AU
Anti- RNP	5.6 AU	<1 AU
C3	55 mg/dL	<84 AU
C4	14 mg/dL	<15 AU

Upon admission, she was mute and unable to provide history. Psychiatry diagnosed catatonia. Brain imaging was unremarkable, but labs revealed persistent anemia and leukopenia. CSF analysis showed mildly elevated protein, normal IgG index, and minimal WBCs. Infectious causes were ruled out. She was started on hydroxychloroquine, mycophenolate mofetil, and a reduced pulse-dose steroid regimen (methylprednisolone 250 mg IV daily). Concomitantly, she was treated for catatonia by psychiatry with IV benzodiazepines but showed no improvement. Electroconvulsive therapy (ECT) was initiated, leading to a dramatic improvement in mental status. She is now managed with mycophenolate (1,000 mg BID), and hydroxychloroquine was discontinued due to congenital retinal abnormalities. Her mental status remains stable without recurrence.

## Discussion

SLE is a chronic autoimmune disease with a variety of manifestations. They can range from cutaneous lesions to musculoskeletal complaints, as well as laboratory abnormalities including cytopenia. In more severe cases, there can be organ involvement such as vasculitis, cardiac involvement, renal involvement, and as in the cases discussed above, there can be neurological manifestations. NPSLE encompasses a broad spectrum of neurological and psychiatric manifestations, affecting both the central and peripheral nervous systems, with studies showing prevalence ranging from 17% to 44.5% [5]. The ACR has identified 19 distinct syndromes associated with NPSLE, including cognitive dysfunction, seizures, psychosis, mood disorders, and cerebrovascular disease [6]. The outcomes of NPSLE are variable, and it can have significant impacts on quality of life and mortality, imparting a considerable economic burden.

**Figure 4:**
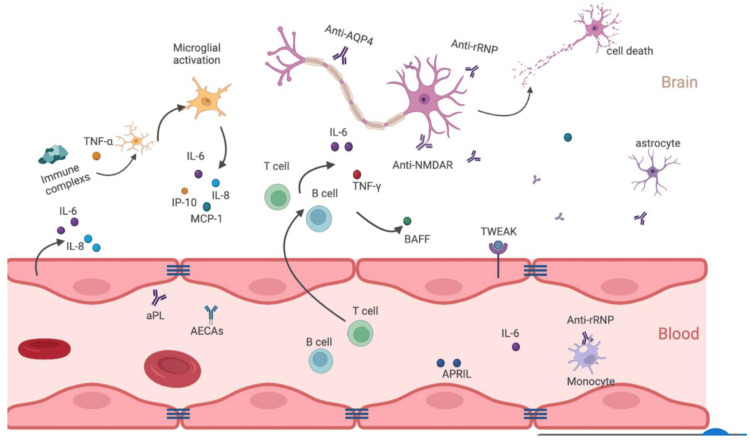
Illustration depicting the pathogenetic mechanisms in diffuse NPSLE where autoimmune-mediated neuroinflammatory and ischemic pathways contribute to the disease. Copyright/License: This figure has been adopted from Wang et al. [8], which is an open-access article distributed under the terms and conditions of the Creative Commons CC BY license.

The pathogenesis of NPSLE is unclear; however, it is believed to be multifactorial, involving ischemic damage, driven by vasculopathy and antiphospholipid antibodies, which can provoke ischemic and embolic events, disruption of the blood-brain barrier [7], autoantibody production, and inflammatory cytokine release. Complement pathways and genetic predispositions are also believed to drive neuroinflammation. Autoantibodies, such as anti-NMDA receptor antibodies, anti-ribosomal P antibodies, anti-endothelial cell antibodies, and anti-ganglioside antibodies, have been implicated in neuronal damage and cognitive dysfunction [8]. Pro-inflammatory cytokines such as interleukin-6 and tumor necrosis factor-alpha contribute to neuro-inflammation and neuronal injury and disrupt the blood-brain barrier [9]. Figure 4 illustrates this proposed multifactorial mechanism of injury.

Diagnosing NPSLE remains complex due to the absence of specific biomarkers and the overlap of symptoms with other neuropsychiatric conditions. The diagnosis of NPSLE largely remains a diagnosis of exclusion. MRI and CSF analysis are the cornerstones of initial evaluation; however, findings are often nonspecific, and none of these tests are definitive. Manifestations of NPSLE can come on acutely, and, while fast action is needed, it is imperative to rule out infection with CSF analysis. Advanced imaging techniques, such as functional MRI and PET, are being explored for their potential to detect subtle brain abnormalities [10].

Management strategies for NPSLE should be tailored to the specific manifestations and severity of the disease. High-dose corticosteroids are the cornerstone of treatment for severe presentations, often in combination with immunosuppressive agents such as cyclophosphamide or mycophenolate mofetil [11]. Severe events such as status epilepticus and transverse myelitis carry a 12.5% one-year mortality, whereas milder syndromes (e.g., idiopathic intracranial hypertension, ataxia) often fully resolve [12]. Novel therapeutic approaches such as Janus Kinase (JAK) inhibitors, interferon receptor antagonists, and complement inhibitors are currently being studied. In less severe cases, treatment can be more symptom-specific, such as anti-epileptics, antipsychotics, anxiolytics, mood-stabilizers, and anti-depressants. In cases refractory to conventional therapy, biologics targeting B cells, such as rituximab, have shown promise [13].

## Conclusions

The cases presented in this series highlight some of the diverse clinical presentations of NPSLE and underscore the importance of a multidisciplinary approach in diagnosis and management. Early recognition and prompt initiation of appropriate therapy are crucial in improving patient outcomes. Ongoing research into the underlying mechanisms of NPSLE and the development of targeted therapies hold promise for enhancing the care of patients with this complex condition.
